# Ultrashort anisoperistaltic end-to-side ureteroureterostomy in renal transplantation

**DOI:** 10.1590/0100-6991e-20223365-en

**Published:** 2022-11-23

**Authors:** CRISTIANO SOUZA LEÃO, RAFAEL AZEVEDO FOINQUINOS, ANA LUIZA DE SOUZA LEAO, ILAN CUBITS KYRILLOS OLIVEIRA CAPELA, MARIA JULIA GONÇALVES MELLO

**Affiliations:** 1 - Instituto de Medicina Integral Prof. Fernando Figueira - IMIP, Clínica Cirúrgica - Recife - PE - Brasil; 2 - Instituto de Medicina Integral Prof. Fernando Figueira - IMIP, Unidade Geral de Transplantes - Recife - PE - Brasil; 3 - Faculdade de Medicina de Olinda - FMO - Recife - PE - Brasil; 4 - Faculdade Pernambucana de Saúde - FPS - Recife - PE - Brasil; 5 - Instituto de Medicina Integral Prof. Fernando Figueira - IMIP, Departamento de Pesquisa e Pós Graduação - Recife - PE - Brasil

**Keywords:** Kidney Transplantation, Urological Surgical Procedures, Surgical Anastomosis, Postoperative Complications, Ureter, Transplante de Rim, Procedimentos Cirúrgicos Urológicos, Anastomose Cirúrgica, Complicações Pós-Operatórias, Ureter

## Abstract

The improvement of surgical techniques in kidney transplantation aims to reduce the incidence of post-transplant complications, contributing to the reduction of hospital stay, related costs, morbidity and mortality, in addition to improving the quality of life of patients. The choice of the best technique is influenced by several factors and the most common technique for urinary tract reconstruction in transplants is performed with implantation of the ureter of the graft in the caudal position, with the anastomosis performed in the bladder. However, the kidney pole can be inverted and the graft ureter anastomosis can be performed directly on the recipient’s ureter, facilitating venous and ureteral anastomoses and reducing urological complications.

## RESUMO

O aprimoramento das técnicas cirúrgicas no transplante renal tem o objetivo de reduzir a incidência de complicações pós-transplante, contribuindo com a redução do tempo de internamento hospitalar, os custos relacionados, a morbidade e a mortalidade, além de melhorar a qualidade de vida dos pacientes. A escolha da melhor técnica é influenciada por diversos fatores e a técnica mais comum de reconstrução do trato urinário nos transplantes é a realizada com implante do ureter do enxerto na posição caudal, com a anastomose feita na bexiga. No entanto, pode-se inverter o polo do rim, posicionando o polo inferior cranialmente e realizar a anastomose do ureter do enxerto diretamente no ureter do receptor, facilitando a anastomose ureteral e diminuindo as complicações urológicas.

## INTRODUCTION

The improvement of surgical techniques and the emergence of more effective immunosuppressive drugs have transformed kidney transplantation into a substitutive therapy with a high quality of life and a significant increase in survival. Despite the gradual reduction in operative morbidity, the procedure involves a wide variety of complex and detailed steps both on graft and its recipient, adding relevant surgical complications, especially those related to the urinary tract^1^. Improvements in suture material and refinement of operative techniques, however, have reduced the incidence of postoperative complications and mortality, also contributing to the reduction of hospitalization time and costs^2^.

As a general rule, the technique usually used consists of retroperitoneal implantation of the graft via an arciform incision in the iliac fossa. Vascular anastomoses are performed in an end-to-side fashion in the external iliac artery and vein^3^.

Historically, the Politano-Leadbetter intravesical ureteroneocystostomy anastomosis was used as the primary technique of ureteral reconstruction in renal transplantation. However, it has fallen out of favor due to high complication rates. Extravesical ureteroneocystostomy techniques, which avoid large openings in the bladder and have shorter operative times, have reduced complication rates from 10% to 6.3%, the Lich-Gregoir technique being the most used today. In these two techniques, the graft ureter is implanted in the usual position, that is, with the lower pole in the caudal position^4^ ^,^ ^5^.

## TECHNIQUE

**Figure 1:**
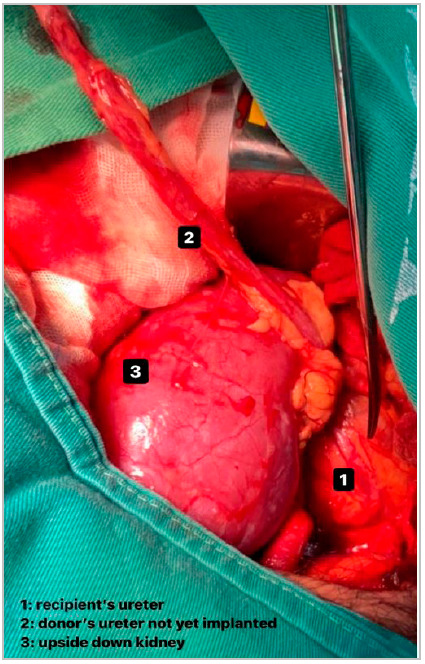
Position of the inverted kidney and its relationship with the ureters.

The operative management of patients, including anesthetic routine, surgical positioning, access, and preparation of the extraperitoneal space to receive the graft are performed in the usual way. In order to use the ultrashort anisoperistaltic end-to-side ureteroureterostomy technique, however, it is necessary to invert the graft, with cranial apposition of the lower renal pole, to bring the structures of the transplanted kidney closer to the homologous structures of the recipient (Figure 1). 

Following the usual sequence of anastomoses in transplants, venous and arterial anastomoses are performed. The renal artery and vein of the graft are anastomosed with the respective recipient’s external iliac artery and vein, using an atraumatic needle and 6-0 polypropylene thread in continuous suture, including all layers of the vascular wall. After graft perfusion, the ureteral anastomosis is carried out.

**Figure 2:**
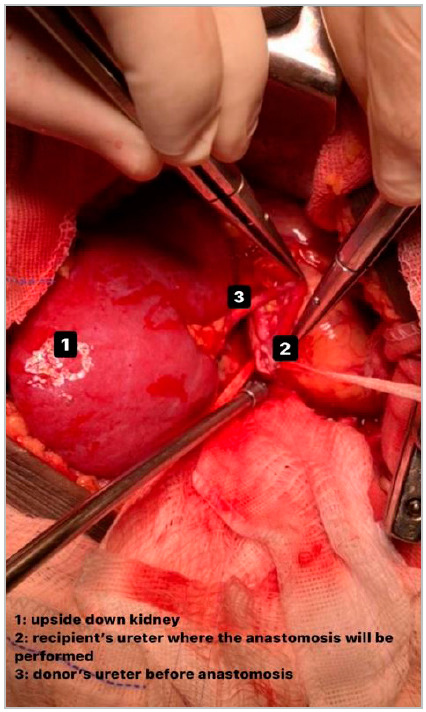
Open recipient ureter before end-to-side anastomosis.

**Figure 3:**
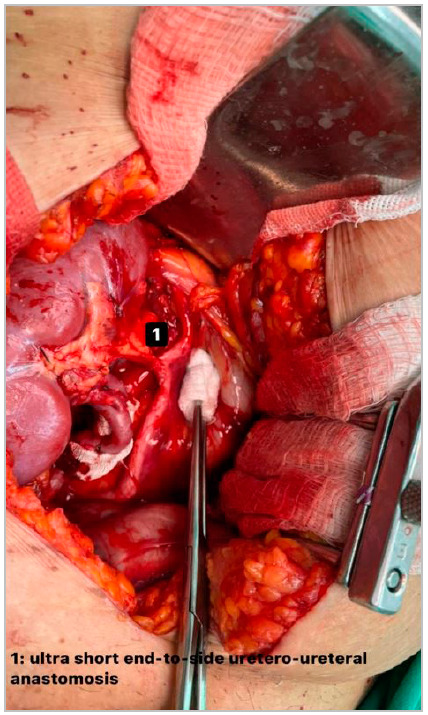
Ultrashort end-to-side ureteroureterostomy.

**Figure 4:**
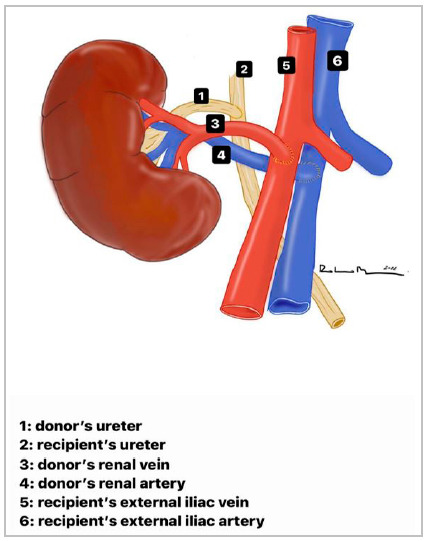
Inverted kidney and its anastomoses.

The graft ureter is positioned cranially and very close to the native ureter, at the level of its intersection with the common iliac artery (Figure 2). This inverted positioning allows an end-to-side ureteroureterostomy with an ultra-short transplanted ureter, approximately 2.0cm, preserving the vast periureteral vascular network (Figure 3). Performing a ureteroureterostomy avoids leaving a long ureter, prone to angulation, ischemia, or necrosis. The suture includes all planes of the ureteral wall and is performed continuously using a 6-0 polydioxanone thread on an atraumatic needle. Finally, revision, synthesis, and postoperative care all proceed in the usual way, even dispensing with the placement of a double-J ureteral catheter and drainage of the surgical bed (Figure 4).

## DISCUSSION

Heterotopic kidney transplantation commonly involves the location of the implant in a craniocaudal position in the recipient’s iliac fossa and the creation of an extravesical ureteroneocystostomy by the Lich-Gregoir technique^5^. In this context, surgical complications may occur in up to 15% of cases, most of which related to the urinary tract (5.9%)^6^. Among the urinary complications, the most common are ureteral stenosis and fistula, with incidences ranging from 2.7% to 6.5% and 1.6% to 5.4%, respectively^7^.

The renal inversion strategy, first described in the early 1970s, is not common and its use raises some questions. One of them is whether this position would cause any problems in the graft’s vascularization or if it would make it difficult to drain the urinary tract, since there would be a need to leave a longer ureter in the case of bladder anastomoses. However, this is not what happens: this approach, inverting the renal poles and positioning the inferior renal pole cranially, brings the urinary structures of the transplanted kidney together with the recipient’s, facilitating the performance of the ultra-short ureteral anastomosis.

Although most kidney transplant groups reserve ureteroureterostomy as a salvage technique for kidney transplants with ureteral complications, few articles in the literature observed a lower rate of reoperation with this technique. One of the explanations is that impaired vascularization of the graft’s distal ureter would be responsible for stenosis and necrosis of the ureter’s tip, with urine leakage^8^ ^,^ ^9^.

A major concern for surgeons who do not perform this anastomosis is how to treat potential ureteral complications, as the native ureter is no longer available for further repair. However, the opposite occurs, since the use of the native ureter allows easy endoscopic catheterization and, therefore, easy access to common endourological procedures, such as ureteroscopy, to investigate or treat any urological event. In addition, despite the anti-reflux system performed in the vesical ureteral reimplantation technique, the incidence of vesicoureteral reflux remains significant and increases the risk of recurrent urinary tract infection and pyelonephritis, consequently impairing graft function^10^.

With the end-to-side ureteroureterostomy technique, the bladder catheter is removed on the first postoperative day, there is a lower risk of urinary tract infection, the placement of a double-J catheter is not necessary, and the endo-ureteral manipulation occurs in a physiological way, similar to non-transplanted patients, reducing the failure related to access when the implant is done in the bladder dome. The ease of this technique reduces surgical time, allows reproducibility among surgeons, and preserves the recipient’s anatomy.
